# Foreword: Towards Accelerating Tool Development for Global Health

**DOI:** 10.4269/ajtmh.22-0114

**Published:** 2022-03-15

**Authors:** Eric A. Ottesen

**Affiliations:** NTD Support Center, Task Force for Global Health, Decatur, Georgia

During the past 75 years, one of the most important, well-appreciated, and enduring achievements of the biomedical research community has been the progressive development of codes and standards for the ethical conduct of research on human beings (individuals or populations) so that new medicines, tools, and strategies to improve human health can be developed, tested, and introduced. Phase 1 research studies in individuals advance through phase 2 and phase 3 before moving into population-wide phase 4 studies—all the while being scrutinized closely by national and international regulatory bodies, and monitored by ethical review boards. The result has been an elegant process in which “business as usual” has led to the development and availability of the safe, high-quality medicines, tools, and strategies that society has come to expect of the biomedical community.

But, this “elegant process” comes at a cost—one that is not just economic, but is also measurable in human suffering. Time is the major culprit here, because the elegant process is a methodical sequence of developmental steps, each determined by results at the preceding step, and often carried out by different cooperating or competing groups. It has been commonly estimated to take more than 20 years to develop a medicine from discovery through successful testing, clinical trials, and ultimate registration for use in people. Shortening this time could be transformational, and in neglected tropical diseases (NTDs), where affected populations number in the hundreds of millions, available interventions (medicines, tools, and strategies) are so few, and economic rewards from new approaches so meager, that progress is typically very slow, with each year lost translating into an enormous burden of human suffering.

What does it take to speed up this process without compromising the scientific rigor and ethical standards required for product development? At one extreme, we have recently witnessed what must be the most dramatic way possible to accelerate this development process. In a truly global response to the COVID-19 pandemic, money, commitment, and human resources became essentially “unlimited” to find solutions to this pandemic. Rather than the usual methodical way in which each programmatic step builds on the results of the previous one, essentially all possible options were pursued at the same time. Those that proved unhelpful were simply discarded—despite their cost—and those that looked promising were explored fully. Unprecedented numbers of researchers, institutions, medical teams, industrial partners, governmental and nongovernmental development organizations, national governments, and international agencies pulled together with a common goal. The result has been the unprecedented, extraordinarily rapid production of diagnostics, vaccines, therapeutics, and deployment strategies now available on a global scale, all generated without compromise of the scientific, ethical, and regulatory standards required today.

But, back in the “real world,” where such mobilization and commitment of resources is impossible for most global health responses, the need to fast-track the creation-to-implementation timeline for new products and strategies remains as serious a problem as ever. Global health equity demands that this timeline be shortened.

Because important examples do exist in which program timelines *have* been shortened successfully, this Supplement to the *American Journal of Tropical Medicine and Hygiene* (“Accelerating Innovation to Impact: Learning from Triple-Drug Therapy [ivermectin, diethylcarbamazine, and albendazole] for Lymphatic Filariasis and from Other Innovations”) was created to highlight key elements of the rationale, organizing principles, and practical lessons learned from these studies that might be emulated usefully elsewhere. Four different kinds of successfully accelerated programs are presented in the Supplement: 1) the multicountry introduction of a new triple-drug treatment approach to eliminating lymphatic filariasis (LF), 2) introduction of a new antiviral vaccine in India, 3) multicountry introduction of a new diagnostic tool for filariasis, and 4) development and introduction of new drug treatment of human African trypanosomiasis.

Introducing the Supplement, Mwele Malecela ^1^ offers a strong reminder of the essentialness of both innovation and the introduction of new tools for the success of WHO global NTD programs during the 2020 to 2030 decade. Considering triple-drug therapy for LF, Julie Jacobson ^2^ lays out ways to bend timelines to accelerate programs from proof-of-concept to implementation into community-wide drug administration programs in endemic countries; Gary Weil et al. present the challenges of assessing the efficacy and tolerability of new ivermectin, diethylcarbamazine, and albendazole regimens for LF both in clinical trials ^3^ and in community settings ^4^; Jonathan King et al. ^5^ discuss the importance of engaging the WHO early in designing the acceleration process; and details surrounding the field implementation and monitoring challenges of ivermectin, diethylcarbamazine, and albendazole introduction are addressed by Bhupendra Tripathi et al. ^6^ for India and by Merelesita Rainima-Qaniuci et al. ^7^ for Pacific island countries. The final three subject articles in the Supplement emphasize the similarity of approaches for accelerating the timeline when introducing a vaccine for Japanese encephalitis to India (Raj Ghosh et al. ^8^), re-tooling and introducing a new point-of-care diagnostic (filariasis test strip) for LF by Anastasia Pantelias et al., ^9^ and the remarkable story from Olaf Valverde et al. ^10^ of developing and introducing fexinidazole for human African trypanosomiasis. Last, Julie Jacobson and Alan Brooks ^11^ draw on the metaphor of creating a symphony orchestra to emphasize the essentialness of mutually beneficial partnerships and enlightened leadership to create an environment in which programs for developing new medicines, tools, and strategies to improve global health can progress as rapidly and efficiently as possible.

The articles in this Supplement give a clear demonstration that accelerated development programs, when carried out responsibly and effectively, have the potential to minimize dramatically the burden of human suffering from NTDs in affected populations. Such acceleration should be facilitated whenever possible to address today’s pressing global health needs.
